# An Agentic Familiarity: The Context of HIV/AIDS and Sexual Orientation for Older Canadians during the COVID-19 Pandemic

**DOI:** 10.3390/healthcare11212869

**Published:** 2023-10-31

**Authors:** Brian de Vries, Gloria Gutman, Robert Beringer, Paneet Gill, Mojgan Karbakhsh

**Affiliations:** 1 Gerontology Program, San Francisco State University, San Francisco, CA 94132, USA; 2 Department of Gerontology/Gerontology Research Centre, Simon Fraser University, Vancouver, BC V6B 5K3, Canada; gutman@sfu.ca (G.G.); robertberinger@uvic.ca (R.B.); paneet_gill@sfu.ca (P.G.); mkarbakh@sfu.ca (M.K.); 3 School of Public Health and Social Policy, University of Victoria, Victoria, BC V8W 2Y2, Canada

**Keywords:** COVID-19, HIV/AIDS, sexual orientation (lesbian; gay; bisexual), future planning

## Abstract

This paper examines how experiences with a previous pandemic, particularly HIV/AIDS, may have informed approaches to COVID-19, with a focus on sexual orientation. Method: The sample was drawn from an online survey of Canadians 55+ conducted in 2020, comprising 1143 persons (mean age = 67; 88 gay or bisexual (GB) men, 65 lesbian or bisexual (LB) women, 818 heterosexual women, and 172 heterosexual men). Respondents reported if they, or someone close to them, “had been affected by” one or more pandemics and whether COVID-19 led them to “think more about their prior epidemic/pandemic experiences” and/or feel they “couldn’t handle it again”. Correlated items reflecting feeling “they have been here before”; “prepared for what is happening”; and “like they needed to act or do something” formed a scale named “agentic familiarity”. Results: About half of respondents reported thinking about their previous pandemic experience; about 5% reporting feeling like “they couldn’t handle it again” with no gender or sexual orientation differences. Higher agentic familiarity scores were found for GB men and for those with experience with HIV/AIDS vs. other pandemics. Discussion: These outcomes speak to resilience and growth experienced by LGBT (and especially GB) persons through shared stigma and trauma—with implications for current pandemic experiences and future actions, like advance care planning.

## 1. Introduction

COVID-19 has often been described as the first pandemic in over a century. In fact, there have been others, including the Spanish Flu of 1918–1920 [1,2], tuberculosis in the late 19th century; polio in the 1950s; SARS in 2002; the H1N1 influenza pandemic in 2009–2010; and HIV/AIDS, first identified in the early 1980s and continuing as a major public health issue. These previous epidemics and pandemics exist as context for many of those now confronting COVID-19.

In this study, we seek to understand this context and its effects, focusing primarily on HIV/AIDS. In 2022, almost 39 million people around the globe were living with HIV/AIDS [3]; in North America, African American and Latinx/Hispanic persons are disproportionately affected by HIV/AIDS relative to other racial groups [4]; however, across all racial and ethnic groups, gay and bisexual men, have been, and continue to be, disproportionately affected by HIV/AIDS—both amongst those newly diagnosed as well as those living with it [5]. Lesbians also have played a significant role in the HIV/AIDS crisis, often being among the first to provide care, when no one else would, in the early years of the epidemic and grieving the loss of many in their networks [6]. Concomitantly, HIV/AIDS has been identified as a pivotal dimension in the lesbian, gay, bisexual and transgender (LGBT) rights movement through community confrontation of both the stigma and discrimination encountered by persons living with HIV/AIDS and the related failure of governments to address the health crisis as it unfolded, resulting in hundreds of thousands of deaths of mostly among gay and bisexual (GB) men and other men who have sex with men—and the millions of deaths of women and men worldwide. HIV/AIDS has taken on social and cultural significance, particularly for LGBT persons, in addition to its physiological manifestations. We propose that these ongoing and contextual experiences impact the ways in which LGBT persons, and especially GB men, approach and experience the COVID-19 pandemic. This impact may take two (or more) forms: one possibility is that LGBT persons may rehash this period and experience coping difficulties with COVID-19; alternatively, LGBT persons may draw from these earlier experiences in an empowering, action-oriented manner.

### 1.1. COVID-19

An impressive literature is emerging on the experiences, lessons learned, and losses of COVID-19. Since it was first declared a pandemic (in March 2020), through its many waves and variants and to the present, COVID-19 has changed lives in myriad ways: through death and illness; the ways and frequency with which people interact with one another; the ways we work and travel; our daily routines; and our goals, hopes, and fears. Globally (as of 9 August 2023), almost 7 million people have died [7], and hundreds of millions of persons have become ill, some continuing to experience symptoms up to three years after first becoming sick. The pandemic has also changed institutions and businesses, counties and countries, and almost every social organization in between.

Several authors have written about the lessons learned through all of this, including the etiology and epidemiology of the virus; modes of transmission and clinical manifestations; pathophysiology, diagnosis, treatment, therapies, and prevention [8]. Almost all publications detailing the learnings from this pandemic highlight previously undervalued areas brought to the foreground, such as the interlinking spheres of health (e.g., physical, economic, public) [9], the importance of attending to mental health, as well as the inequalities and inequities revealed through the pandemic [10], both across and within countries [11]. It became clear that age was a key source of inequality: those aged 65 and older had mortality rates at least ten times higher than those younger than 40 [11]. Other areas of inequality included race, those with pre-existing conditions, the immunocompromised, and persons living with physical or mental disabilities. Andrasik et al. [12] write that: “For most infectious diseases, including COVID-19, the most extreme burden of disease is experienced by society’s most vulnerable, most often, people who experience multiple forms of social disadvantage” (p. 297). 

### 1.2. LGBT Persons and COVID-19 

LGBT older adults are among these vulnerable adults with, relative to the general population, poorer physical and mental health, higher rates of health risk behavior, higher rates of singlehood and living alone, loneliness, fewer identified caregivers, and lower rates of socio-economic status—all directly or indirectly related to higher rates of social stigma, discrimination, and victimization [13,14]. As Gibb et al. [15] note, LGBT persons have both biocultural and social vulnerabilities potentially exacerbated by COVID-19 as well as potential resilience and protective factors that may be energized by the pandemic; resilience has emerged as a dominant theme in recent research on LGBT older adults [16]. Along the lines of the former, Fish et al. [17] found significantly lower levels of physical and mental health pre-pandemic and greater declines in these measures post-pandemic for gay men as well as bisexual men and women. Kneale and Bécares [18] similarly found high levels of stress and depressive symptoms among their LGBT sample during the early months of the pandemic—and higher than what was observed in previous research and with other marginalized samples in this study; the scores were particularly elevated for transgender and gender non-conforming adults, and younger persons. 

Bouton et al. [19] noted greater anxiety and symptoms of depression among both younger and older LGBT adults, compared to similarly aged straight, cisgender persons during the first year of the pandemic. LGBT persons, of both age cohorts, were also more likely to seek therapy and to be prescribed medication for their mental health. At the same time, LGBT persons were slightly more likely to have received a COVID vaccine. Gutman et al. [20] reported results along similar dimensions in their large study of older Canadians and their experiences during the pandemic, upon which this present study is based. That is, they found that, relative to heterosexuals in the sample, LGBT persons were more likely to report feeling anxious, depressed, and sad and more likely to report changes in access to their physical and mental healthcare. However, LGBT persons were also significantly more likely to adhere to public health orders and take protective action (i.e., wear masks, social distance, restrict social engagements) than were heterosexual persons. 

These latter two studies, especially, highlight both the vulnerabilities as well as the resilience and protective behaviors of LGBT persons during the COVID pandemic to which Gibb et al. [15] directed our attention. Multiple authors refer to the HIV/AIDS crisis in their analyses of these responses to the COVID-19 pandemic by LGBT persons [18,20]—drawing attention to previous epidemic experiences, and their concomitant risks and defenses, vulnerabilities, and protections. 

### 1.3. COVID-19 and HIV/AIDS

Early in the COVID-19 pandemic, some in the popular media offered comparisons between the COVID-19 and the HIV/AIDS pandemic, both how the viruses and pandemics differed as well as their parallels—particularly in the socio-cultural experiences, and especially for sexual and gender minority persons. Notwithstanding that the COVID-19 virus has biochemically more in common with previous SARS and even H1N1 viruses than HIV/AIDS and that HIV/AIDS is more difficult to transmit and slower to manifest and inflict illness, the pandemic that COVID-19 has created is more reminiscent of the (especially) early years of HIV/AIDS. During those years in the early 1980s, there were suspicion and acts of violence (mostly against GB men and men who have sex with men), discussions of quarantines and stigma of those infected believed to be carriers, and shifting public health messages as officials learned more and searched for ways to find a message that reached intended audiences. These experiences of suspicion, stigma, and changing public discourse resonate strongly with older LGBT persons and especially GB men, many of whom “came of age” during these early years of the epidemic, eponymously labeled early on as Gay-Related Immune Deficiency (GRID) (and colloquially as the “gay plague”) [21]. As Richard Berkowtiz, a well-known US AIDS activist, was quoted as saying in the New York Times: “Wow! Lucky me. I actually managed to survive one pandemic to be here for another one” [22] (para. 48). 

The same article quoted several other well-known gay men describing their current experiences with COVID-19, carrying on the vulnerability and resilience duality. Peter Staley, for example, another prominent US AIDS activist, was quoted as saying: “there is no denying that for me and other long-term survivors of the AIDS crisis I know, COVID-19 is stirring up a lot. To the extent that all of us from those years have some version of PTSD, all of that is flooding back” [22] (para. 10). In contrast to this return of trauma, writer Hal Rubenstein offered a protection and resilience narrative: “I do think I’ve learned from HIV not to be foolish. I do think I’ve learned that if someone else isn’t going to watch out for me, then I’ll watch out for myself” [22] (para. 42). 

Scholarly literature has reinforced this duality. Several qualitative studies have explored the approach taken to the COVID-19 pandemic by LGBT persons, mostly gay men, with HIV/AIDS as a backdrop. Handlovsky et al. [23] interviewed older gay men and explicitly juxtaposed experiences during the present pandemic with the HIV/AIDS pandemic. The themes emerging from these considerations highlighted a sense of pandemic familiarity with associated distress and challenge (one participant describing being forced back into a (different) closet); respondents also contrasted the early lack of response to HIV/AIDS to the accelerated response to COVID-19, and the difference in the speed and spread of information between the two pandemics, rekindling some anger and frustration, but also some satisfaction in what had been accomplished and what had been learned. Quinn et al. [24] found that about one-third (35%) of their sample of gender minority men reported that HIV/AIDS helped them cope with COVID-19. In coded, open-ended responses, the men offered four themes: experience having lived through a pandemic, experience coping with stigma, personal responsibility, and belief in collective action. Stigma and resilience ran through these themes. Gonzalez et al. [16] presented the narrative analyses of LGBT adults ranging in ages from 19 to 75 focusing on resilience in the time of COVID-19. Three broader themes were presented: preparation fostered by resilience (including experiences with isolation, marginalization, and HIV/AIDS); radical acceptance as resilience (including acceptance of oneself, reality, privilege, and responsibility); and resilience through support and community building. 

### 1.4. The Present Study

Both the popular and scholarly literatures point to a duality of COVID-19 perspectives and experience by LGBT persons as influenced by their previous experiences with HIV/AIDS. We propose that these connections are most strongly experienced by older GB men and that these connections may manifest in multiple ways. For example, LGBT persons, and especially GB men, may think of their previous (HIV/AIDS) pandemic experiences more often than other groups, and along with these recollections of previous experiences, they may feel either prepared/drawn to action by the current pandemic or overwhelmed/unable to handle this crisis. We tested these competing hypotheses (i.e., previous pandemic experiences, and particularly HIV/AIDS, may innervate or bolster respondents or may enervate or overwhelm respondents), drawing from our national study of older persons and their experience of the COVID-19 pandemic. 

## 2. Materials and Methods

This study is based upon an online survey of persons aged 55 and over living in Canada and focused on current experiences and future plans during the COVID-19 pandemic. Specifically, the invitation described the purpose of our survey as exploring “any pandemic-related stressors you may be experiencing, issues you are facing regarding healthcare, and any actions taken toward planning for future care”. We further mentioned that we were seeking respondents from the general population as well as targeting responses from minority groups, including those who self-identify as LGBT. Potential respondents were informed, on the consent page, that no payment was to be provided, the rights of the research participant, including anonymity, and an email and phone contact if one had questions about their participation and/or the study. The survey was approved by Simon Fraser University’s Research Ethics Review Board.

The survey questions were developed by the study authors and informed by our work as members of The Diversity Access Team (DAT), which is part of a large pan-Canadian advanced care planning study; the DAT is focused on assessing, tailoring, implementing, and evaluating advance care planning tools aimed at minority populations (specifically, LGBT, South Asian, and Chinese). The survey included basic demographic information and a series of items that concerned health and functional status, pandemic-related stressors and social impacts, healthcare access, and planning for the future. The 61-item survey opened on 10 August 2020 and closed on 10 October 2020 (a PDF copy of the survey is available online at https://www.sfu.ca/lgbteol.html (accessed on 28 October 2023). The mean time spent to complete the survey was just under 14 min.

Respondents were recruited using social media, direct email, and Facebook advertising as well as a comprehensive email campaign targeting organizations serving older adults in general and those with connections to minority older adults, including LGBT persons. Over 80 regional and/or national organizations assisted in promoting the study. The total sample size was 4380 persons; we used Facebook advertising to recruit most of our survey respondents (3240 responses were collected from social media ads). We chose Facebook knowing that 68% of US adults use Facebook and of those, 74% of users access it daily, and it is a medium accessed by all ages including older adults [25].

### Measures

The analyses reported here utilized the following measures: age; education (high school or less, certificate/diploma, undergraduate degree; graduate degree); ethnic, cultural, and racial background (white; BIPOC [Black, Indigenous, People of Color]); sexual orientation (heterosexual; lesbian or gay; bisexual (all with definitions provided), and a “don’t know/no answer” option); gender identity (choosing from man, woman, non-binary, or another gender category of their wording); relationship status (single—never married, married or living as married, widowed, divorced, separated); and living arrangement (living alone or with another). We also asked about transgender identity (yes or no), although numbers were too small for inclusion as a unique category in the analyses. Instead, transgender persons who identified as either men or women were included in those gender categories, and further by identified sexual orientation. 

Respondents were asked if they “or someone close to [them] were affected by one or more of the following pandemics”. The options from which they could choose included “Spanish flu; TB; Polio; HIV/AIDS; SARS; H1N1 flu”; and “other” in which they could write in a response. If respondents selected at least one of the previous pandemic options, including “other”, they were directed to five additional questions about their responses to the COVID-19 pandemic in light of their prior experiences. These questions asked if “Since the COVID-19 pandemic began” they “thought more about that previous pandemic experience” (subsequently referred to as “thought”) with 5-point response scales ranging from (1) “a lot less than usual” through (3) “the same amount as usual” to (5) “a lot more than usual”. Four additional items asked if they felt like they “had been here before” (hereafter referred to as “experienced”) “prepared for what is happening” (“preparation”) needed to “act/do something” (“action”), and “can’t handle it again” (“handle”). For these four items, 5-point response options ranged from (1) “very strongly disagree” through (3) “neutral” to (5) “very strongly agree”. 

Correlations among these five items were calculated and ranged from 0.131 to 0.567, as reported in Table 1. The three highly correlated items were treated as a scale (“experienced”, “preparation”, and “action”) for which Cronbach’s alpha was found to be 0.78. This 3-item scale was labeled as “agentic familiarity” (with values ranging from 3–15) to reflect respondents’ call to action based on experience and understanding (and evoking the theme of resilience found in the literature on LGBT older adults) and was included in the analyses to follow, along with “thought” and “handle” as single items.

## 3. Results

### 3.1. Analytic Sample Characteristics

Our analytic sample comprised those respondents who reported that they or someone close to them had been “affected by one or more” of the listed pandemics, or one not listed but identified by them. In total, 1180 individuals responded affirmatively. The final sample was 1143 (26% of the total sample). Small subsample sizes unfortunately meant, for analytic reasons, that we had to exclude some groups of participants from the analyses; these include those who responded “don’t know, no answer” to the sexual orientation question (n = 23), as well as those who identified as non-binary or an “additional gender category” (n = 8). Additionally, again owing to their small sample size, bisexual women (n = 20) were grouped with lesbians, and bisexual men (n = 4) with gay men for the analyses. 

As can be seen in Table 2, the average age of the analytic sample was 67. LGBT respondents tended to be younger and more likely to be single and to live alone. LGBT persons were also more likely to have a graduate degree. Of those for whom race data were available, 89% identified as “white”.

### 3.2. Pandemic Experience

Overall, H1N1 was the previous pandemic identified most often, followed by polio, both identified by more than 30% of respondents (see Table 3). TB, HIV, and SARS were the next most frequently identified pandemics, all mentioned by between 18 and 20% of respondents; the Spanish Flu was mentioned by more than 12% of respondents. Just over 5% of respondents identified a pandemic not on the list provided; these included multiple mentions of Hong Kong Flu, the Norwalk virus, cancer, hepatitis C, scarlet fever, and the West Nile virus. Unique options were also provided by respondents who entered answers in response to the final probe in the question: “others, please specify”. 

LGBT respondents were significantly more likely to identify HIV/AIDS; heterosexual persons were significantly more likely to identify H1N1, SARS, polio, and the Spanish Flu. This pattern was replicated in comparisons of GB and heterosexual men; for women, apart from the sexual orientation difference in the identification of HIV, the only significant difference was that heterosexual women were *less* likely to identify H1N1 than were LB women. Although not presented in the table, 20.5% (n = 18) of GB men reported that they had previously been diagnosed with HIV/AIDS; one heterosexual man also reported an HIV/AIDS diagnosis. No women reported such a diagnosis. 

A mean of 1.4 previous pandemics were experienced by this sample, slightly higher for women (1.44) than for men (1.43) (F(1,1123) = 5.668, *p* < 0.05). This was qualified, however, by the interaction between sexual orientation and gender (F(1,1123) = 8.358, *p* < 0.005): heterosexual men did not differ in average number of pandemics from GB men, whereas lesbian and bisexual women reported a greater number of pandemics (1.77) than heterosexual women (1.42). 

### 3.3. Hypothesis Testing

Given the ordinal nature of the “thought” and “handle” variables, separate logistic regressions were conducted with the dependent variables dichotomized into “the same or less” vs. “somewhat or more” (in the case of “thought”) and “neutral or disagree” vs. “agree, including strongly” (in the case of “handle”). Age, gender, sexual orientation, having experienced the HIV pandemic, race (white/non-white), living alone, and education, as well as the interaction term between gender and sexual orientation were entered as predictors; both regression equations were non-significant. Almost half (48%, n = 566) of those with previous pandemic experience reported “having thought about” this previous experience at least somewhat more than usual, non-significantly different across groups. Only 5.2% (n = 61) either agreed or strongly agreed with the statement that they felt like they “can’t handle it again” given their previous pandemic experiences, again non-significantly different across groups.

A multiple linear regression was conducted to predict agentic familiarity based on the same predictor variables as noted above. A significant regression equation was found (F(8,1084) = 7.649, *p* < 0.001) with an R^2^ of 0.053. Significant effects were found for having experienced the HIV pandemic as well as the interaction between gender and sexual orientation (see Table 4). In the case of the former, those who had previously experienced the HIV pandemic (vs. other pandemics) had higher scores on the agentic familiarity measure, 8.90 vs. 7.85 (F(1,1069) = 14.826, *p* < 0.001). In the case of the latter, GB men had higher scores than any of the other groups. Scores were as follows: GB men: 9.67; heterosexual men: 8.13, lesbian and bisexual women: 7.92; and heterosexual women: 7.79 (F(1,1069) = 3.942, *p* < 0.05). 

## 4. Discussion

Notwithstanding claims of COVID-19 being the first pandemic in a century, over one quarter of our national Canadian sample reported having been “affected by one or more” previous pandemics; these included, in descending order of prevalence: H1N1, polio, TB, HIV/AIDS, SARS, the Spanish Flu, and others. Many noted that their experiences included more than one previous pandemic (the average number of pandemics was 1.4). The particular experience of each of the pandemics undoubtedly varied; for example, it was likely someone other than the survey respondent was personally affected by the Spanish flu, given both the age of respondents and the time of the Spanish flu. Presumably, other pandemics were more personally experienced; we know this to be the case with HIV/AIDS for whom over 20% of the gay and bisexual men in our sample had received such a diagnosis.

Almost half of the respondents thought about their previous pandemic experiences during the COVID-19 pandemic, independent of which previous pandemic experience they had, though very few (just over 5%) felt that they “couldn’t handle it” again. On neither of these measures was there a significant sexual orientation difference, disconfirming one of our competing hypotheses. HIV/AIDS is the previous pandemic most associated with feeling experienced, prepared, and needing to take action (i.e., agentic familiarity) in the current context—and GB men had the highest scores on this same measure. 

These two findings are linked. In North America, HIV/AIDS was initially (and remains) strongly associated with a marginalized group: men who have sex with men. In the early years of the HIV/AIDS pandemic, images and news about the virus and the mostly GB men living with it—but mostly dying from it—were poignantly and abundantly present; at the same time, this virus and affected groups were either ignored by governments or attacked/blamed for what was happening, often by religious leaders [26]. Instead of allowing HIV and those living with it to die in obscurity (which some believed was the unspoken government strategy), government non-response and religious rebuke motivated the development of a strong, vocal, caring, and active LGBT community. Perhaps more so than any other virus and/or pandemic (potentially excepting COVID-19), HIV/AIDS became political—so did LGBT communities. 

This is part of the backstory of the well-publicized actions undertaken by LGBT persons and allies during the early years of the HIV/AIDS crisis, including: the formation of ACT-UP (the AIDS Coalition to Unleash Power) and other national and international protest movements; pressuring the US Centers for Disease Control to change the way drugs are tested and brought into market; creating myriad organizations to serve the needs of those who were sick with and dying from this virus [27]. These are legacies of social justice and changed social structures and attitudes [28]. As such, HIV/AIDS is part of the social cultural fabric of life for older LGBT persons, and especially GB men. Recall that almost 88% of GB men report having been “affected by” HIV/AIDS—more than four times the number of respondents who report having been diagnosed with HIV/AIDS. Gorman and Nelson [29] wrote that almost every older “gay person … alive today has been touched by the AIDS epidemic in one form or another” (p. 79). 

We propose that it is this social cultural experience, this prior preparation and previous action that is carried forward from HIV/AIDS and seen in COVID-19 agentic familiarity, especially by GB men (consistent with the competing hypothesis). This is also consistent with the research findings of Quinn et al. [24] (e.g., the experience of having lived through the HIV/AIDS epidemic enhanced coping with COVID-19) and the experience and acceptance themes of resilience described by Gonzalez et al. [16] as reported by LGBT persons. Bouton et al. [19] note that LGBT persons were somewhat more likely to have received COVID-19 vaccines than were heterosexual persons, taking their HIV/AIDS lessons and applying them to COVID-19. These results may also be seen in the context of resilience and hardiness on the part of LGBT persons, often reported in recent literature [30]. 

As noted above, Gutman et al. [20] extended these findings even further into the responses of LGBT persons (all over the age of 55) to the COVID-19 pandemic. They found, for example, that LGBT persons were more likely than were cisgender, heterosexual persons to wear masks, to social distance, and to follow other public health mandates proclaimed during the COVID-19 pandemic. They suggested that LGBT persons took the pandemic more seriously and acted with intention—with some personal (e.g., greater changes to access to healthcare) and mental health costs in so doing (e.g., higher rates of feeling depressed, anxious, and sad). Our findings add further depth to these findings of Gutman et al. [20]: these adherence behaviors are likely more than model citizenship or prosocial behaviors; they represent the lessons learned from their previous pandemic and related experiences. 

### 4.1. Implications for End-of-Life Care

These results have implications beyond how LGBT persons, and especially GB men, adhere to public health mandates and approach the COVID-19 pandemic. de Vries et al. [31], for example, make inferences to these same phenomena in their analyses of Advance Care Planning (ACP) among older LGBT persons, drawing from the same sample as the present study. They found that since the onset of the COVID-19 pandemic, LGBT persons were 1.6 times more likely than were cisgender heterosexual persons of comparable age to have begun or completed at least one of the following: a will, durable power of attorney, Advance Directives, and/or a Representation Agreement. So, too, were LGBT persons more likely to have had care discussions since the start of the pandemic—1.5 times more likely. The results of the present study further inform the link between sexual orientation and ACP. These planning behaviors by LGBT persons may be seen as examples of pandemic-related agentic familiarity: feeling the need to “act/do something” which in this instance meant being “prepared for what is happening” given that these individuals “had been here before”. 

Even prior to the pandemic, however, a greater number of LGBT persons (than heterosexual persons) reported having an Advance Directive and having engaged in an end-of-life conversation—with their spouses/partners (if in a relationship) (74% vs. 57%), with their friends (22% vs. 11%), or with their health care provider (though these latter conversations were surprisingly rare—about 7% vs. 4%) [31]. These results too have been interpreted as falling within the shadow cast by HIV/AIDS in the lives of older LGBT persons, and especially GB men: most older gay (and bisexual) men know of someone, and many have cared for someone, who died of HIV/AIDS [29]—often more than one. In previous research, we have found [32] that more than one quarter of our sample of middle-aged and older gay men reported having lost 15 or more friends to HIV/AIDS. There may be an awareness of end of life and death unlike that seen among many other groups of people (with potential exceptions like battlefront or other such frontline veterans of death)—motivating action. 

This awareness and deep knowledge are both fueled and challenged by a legacy of stigma, discrimination, and marginalization. Today’s older LGBT adults were labeled as sick during their youth, by the same medical system to whom they must now turn for healthcare support, hence many are naturally suspicious of this medical environment [33]. Policies that prohibited marriage and discounted same-sex relationships led to higher rates of singlehood, childlessness, and living alone, especially among older gay men [13]; many older gay men receive insufficient support [34], and/or are without caregivers. Many gay men learned they could only rely on themselves—only realizing another side of self-sufficiency in later life; a colleague once remarked, “growing up, I had to be fiercely independent as a gay man; as a senior, I became severely isolated” (Richard Bass, personal communication, 7 April 2023). 

These are the weights to balance in addressing end-of-life care for older LGBT persons: awareness and preparation with wariness and isolation. Acquaviva [35] thoroughly details the personal, organizational, and institutional reflections needed to provide compassionate, inclusive, thoughtful care to LGBT persons at the end of life. These reflective efforts provide the possibility of rendering care more person-centered, recognizing the history, biography, and context of the person for whom care is offered—efforts important beyond working with LGBT persons, but especially relevant in the framework of this paper. 

### 4.2. Summary and Limitations

This study, with a large and national sample of Canadian older adults, focused on how previous pandemic experiences, particularly HIV/AIDS, may impact the approach taken to COVID-19. Of special interest were the experiences of GB men, a group disproportionately affected by HIV/AIDS; these experiences were thought to either enervate or innervate their responses and approach to COVID-19. Results reveal that HIV/AIDS was the pandemic most strongly associated with an innervating approach, an approach especially noted among GB men and believed to be tied to their cultural and biographical experiences. 

The construct of agentic familiarity emerging in these analyses may have use and applications in research and practice beyond our study. This may include, for example, novel perspectives on locus of control and health in general [36] and/or contributions to the literature on resilience, crisis competence, positive marginality, and aging among LGBT persons [37]. Further foundational work is necessary, however, particularly given the relatively high standard error found in these analyses which limit the precision and reliability of the measures. 

More generally, the results presented above must be understood within the limitations of this exploratory study. The primary question from which previous pandemic experience was determined could have been more direct (i.e., specifying the individual) and focused (i.e., specifying the effect); recall that we asked if you “or someone close to you were affected by one or more of the following pandemics”. Most of the survey items were created based on our review of the (at the time) limited research dealing with COVID-19, findings from focus groups conducted for related studies, and personal experience—constructed in a tight time frame to launch the survey early on in the pandemic. Even as the survey was pilot tested with a small group of respondents meeting the eligibility criteria, and modest revisions were made, the reliability and validity of these instruments are largely unknown. Recruiting for an online survey particularly among older adults and using Facebook as a pivotal source may have contributed to the larger number of women in our sample—and other unknown unique features of the sample compromising representativeness. Sample sizes are so often an issue, even within a large, national sample, when it comes to stigmatized and marginalized minority groups; the sample sizes here meant GB men, and lesbians and bisexual women, were joined for analyses which, even as consistent with previous research, precluded finer level distinctions and experiences. Similarly, the numbers of transgender and gender non-conforming persons were too small to treat independently. Still, these data offer an interesting and telling vantage point for the consideration of history and context in the experience of the COVID-19 pandemic for LGBT older Canadians.

## Figures and Tables

**Table 1:** Correlations among previous pandemic evaluation items (n = 1180).

	“Thought”	“Experienced”	“Preparation”	“Action”	“Handle”
“Thought”		0.326 **	0.137 **	0.291 **	0.177 **
“Experienced”			0.567 **	0.559 **	0.313 **
“Preparation”				0.525 **	0.131 **
“Action”					0.306 **
“Handle”					

** *p* < 0.001.

**Table 2:** Socio-demographic characteristics by gender and sexual orientation.

	Gender						Total	Sexual Orientation	
	Women			Men					
	Heterosexual Women	Lesbian & Bisexual Women	Women Total	Heterosexual Men	Gay & Bisexual Men	Men Total		Heterosexual	LGB
N	818	65	883	172	88	260	1143	990	153
Age (mean)	67.0	64.9	66.9	68.1	66.7	67.6 *	67.0	67.2 *	65.9
Relationship Status (%)									
Single	7.1	24.6 **	8.4	5.2	21.6 **	10.8	8.9	6.8	22.9 **
Married	59.7 **	29.2	57.4	76.7 **	50.0	67.7	59.8	62.6 **	41.2
Widowed	13.6	16.9	13.8	4.1	8.0	5.4	11.9	11.9	11.8
Divorced/Separated	19.7	29.2	20.4	14.0	20.5	16.2	19.4	18.7	24.2
Living Arrangement (%)									
Alone	31.3	53.8 **	33.0	18.6	35.2 *	24.2	31.0	29.1	43.1 **
Education (%)									
High School or less	13.7	13.8	13.7	12.2	19.3	14.6	13.9	13.4	17.0
Vocational College	34.8	30.8	34.5	36.0 *	20.5	30.8	33.7	35.1 *	24.8
Bachelor’s degree	24.8	18.5	24.3	18.6	23.9	20.4	23.4	23.7	21.6
Graduate degree	23.1	35.4 *	24.0	25.6	28.4	26.5	24.6	23.5	31.4 *

* *p* < 0.05; ** *p* < 0.001.

**Table 3:** List of previous pandemics (including multiple responses) by gender and sexual orientation.

	Gender						Sample Total	Sexual Orientation	
	Women			Men					
	Heterosexual Women	Lesbian & Bisexual Women	Women Total	Heterosexual Men	Gay & Bisexual Men	Men Total		Heterosexual	LGB
Spanish Flu	13.6 (111)	13.8 (9) ~	13.6 (120)	12.8 (22) *	3.4 (3)	9.6 (25)	12.5 (145)	13.4 (133) *	7.3 (12)
TB	18.5 (151)	26.2 (17)	19.0 (168)	30.2 (52) **	10.2 (9)	23.4 (61)	20.0 (229)	20.6 (203)	16.5 (26)
Polio	32.0 (262)	26.2 (17)	31.6 (279)	33.1 (57) **	13.6 (12)	26.5 (69)	30.5 (348)	32.2 (319) *	19.5 (29)
HIV/AIDS	13.4 (110)	43.1 (28) **	15.6 (138)	7.0 (12)	87.5 (77) **	34.2 (89) **	19.8 (227)	12.3 (122)	68.9 (105) **
SARS	19.7 (161)	20.0 (13)	19.7 (174)	19.2 (33) *	8.0 (7)	15.4 (40)	18.6 (214)	19.5 (194) *	12.8 (20)
H1N1	37.2 (304) **	43.1 (28)	37.6 (332)	34.9 (60) **	11.4 (10)	26.9 (70)	35.3 (402)	36.6 (364) **	24.4 (38)
Other: respondent-provided - Hong Kong Flu (19) - Norwalk virus (3) - Cancer (2) - Hep C (2) - Scarlet fever (2) - West Nile virus (2) - Others, including Lyme disease, mad cow, MERS, smallpox, typhoid, “toxic liberalism”, measles, mesothelioma	6.2 (51)	1.5 (1)	5.8 (52)	4.7 (8)	3.4 (3)	4.2 (11)	5.4 (63)	6.1 (59)	2.4 (4)

* *p* < 0.05; ** *p* < 0.005; ~ *p* < 0.05 (insufficient cell size for reliable analysis). n = 1092.

**Table 4:** Multiple regression model predicting agentic familiarity.

Predictors	Unstandardized Coefficients		Standardized Coefficient (Beta)	t	*p* Value
	B	Std. Error			
(Constant)	6.433	0.763		8.434	0.000
Age	0.017	0.011	0.047	1.535	0.125
Gender (Male = 1/Female = 0)	0.397	0.231	0.061	1.722	0.085
Sexual orientation (LGBT = 1, Heterosexual = 0)	−0.140	0.350	−0.018	−0.401	0.689
Gender × Sexual orientation	1.063	0.509	0.104	2.086	0.037 ^◊^
Experienced HIV pandemic (HIV = 1/Others = 0)	0.765	0.237	0.113	3.225	0.001 ^◊^
Race (White = 1, Non-white = 0)	−0.333	0.254	−0.039	−1.312	0.190
Living alone (Yes = 1/No = 0)	0.260	0.179	0.045	1.457	0.145
Education ^¶^	0.126	0.078	0.048	1.615	0.107

◊ statistically significant; ¶ Categories included high school or lower, vocational college, Bachelor’s degree, university degree or above; n = 1092.

## Data Availability

The data presented in this study are available on request from the corresponding author. The data are not publicly available due to research ethics board guidelines.
